# Correction for Liu et al., “The imbalance of pulmonary Th17/Treg cells in BALB/c suckling mice infected with respiratory syncytial virus-mediated intestinal immune damage and gut microbiota changes”

**DOI:** 10.1128/spectrum.01812-24

**Published:** 2024-08-29

**Authors:** Jiling Liu, Yixuan Huang, Nian Liu, Huan Qiu, Xiaoyan Zhang, Xiaojie Liu, Maozhang He, Mingwei Chen, Shenghai Huang

## AUTHOR CORRECTION

Volume 12, no. 6, e03283-23, 2024, https://journals.asm.org/doi/10.1128/spectrum.03283-23. The Acknowledgments should read as shown in this author correction.

This study was supported by the National Natural Science Foundation of China (No. 81974306, Shenghai Huang), the National Natural Science Foundation of China (No. 82302568 ; Maozhang He), Major Project of Natural Science Research of Anhui Education Department (No. KJ2019ZD23, Shenghai Huang), Research Fund of Anhui Institute of translational medicine (No. 2017zhyx26, 2021zhyx-B07, Shenghai Huang), National First-Class Undergraduate Program Construction Point (Biotechnology)(Shenghai Huang), and Projects of Education Quality Engineering of Anhui Province (No. 2021jcxkpy013, Shenghai Huang).

